# Association of the Charlson index with risk classification, clinical aspects, and emergency outcomes*

**DOI:** 10.1590/1980-220X-REEUSP-2020-0162

**Published:** 2022-01-24

**Authors:** Ana Paula Santos de Jesus, Meiry Fernanda Pinto Okuno, Cassia Regina Vancini Campanharo, Maria Carolina Barbosa Teixeira Lopes, Ruth Ester Assayag Batista

**Affiliations:** 1 Universidade Federal do Recôncavo da Bahia, Centro de Ciências da Saúde, Santo Antônio de Jesus, BA, Brazil.; 2 Universidade Federal de São Paulo, Escola Paulista de Enfermagem, São Paulo, SP, Brazil.

**Keywords:** Triage, Emergency Medical Services, Epidemiology, Comorbidity, Hospital Mortality, Triagem, Serviços Médicos de Emergência, Epidemiologia, Comorbidade, Mortalidade Hospitalar, Triaje, Servicios Médicos de Urgencia, Epidemiología; Comorbilidad, Mortalidad Hospitalaria

## Abstract

**Objective:**

To exam the association of the age-adjusted Charlson comorbidity index with the categories of risk classification, the clinical aspects, and the patient outcomes in the emergency department.

**Method:**

Cross-sectional, analytical study that analyzed the medical records of 3,624 patients seen in the emergency department. Charlson index scores greater than 2 showed a high rate of comorbidity (mortality risk). T-test and analysis of variance were applied in the analyses.

**Results:**

There was a significant difference between the Charlson comorbidity index and the risk classification, with higher scores found in patients classified in the white (2.57) and red (2.06) categories. Patients with vascular, endocrine, neurological, cardiologic, or device problems, and those who underwent a head tomography had a high rate of comorbidity. In addition, those admitted, transferred, or who died in the emergency room had significantly higher index scores compared to those who were discharged from the hospital.

**Conclusion:**

The high rate of comorbidity was associated with the categories of risk classification, main and nonspecific complaints, performance of a head tomography, and patient outcomes in the emergency room.

## RESUMO

**Objetivo:**

Averiguar la asociación entre el índice de comorbidad de Charlson ajustado a la edad y las categorías de clasificación de riesgo, aspectos clínicos y resultados de pacientes en servicios de urgencias.

**Método:**

Estudio transversal, analítico que analizó la historia clínica de 3.624 pacientes atendidos en urgencias. Escores del índice de Charlson superiores a 2 demostraron alto índice de comorbidad (riesgo de mortalidad). Prueba T y análisis de varianza se aplicaron en los análisis.

**Resultados:**

Hubo una diferencia significativa entre el índice de comorbidad de Charlson y la clasificación de riesgo, con escores superiores encontrados entre los pacientes de la categoría blanca (2,57) y roja (2,06). Pacientes con quejas vasculares, endocrinas, neurológicas, cardiológicas o con problemas en dispositivos, y los que realizaron tomografía de cráneo presentaron alto índice de comorbidad. Además, los ingresados transferidos o los que murieron en urgencias presentaron escores bastante superiores del índice en comparación a los que tuvieron alta.

**Conclusión:**

El alto índice de comorbidad estuvo relacionado con las categorías de la clasificación de riesgo, quejas principales e inespecíficas, realización de tomografía de cráneo y resultados de pacientes en urgencias.

## RESUMEN

**Objetivo:**

Verificar a associação entre o índice de comorbidade de Charlson ajustado à idade e as categorias de classificação de risco, os aspectos clínicos e os desfechos de pacientes no serviço de emergência.

**Método:**

Estudo transversal, analítico que analisou prontuários de 3.624 pacientes atendidos na emergência. Escores do índice de Charlson superiores a 2 retrataram alto índice de comorbidade (risco de mortalidade). Teste t e análise de variância foram aplicados nas análises.

**Resultados:**

Houve diferença significativa entre o índice de comorbidade de Charlson e a classificação de risco, com maiores escores encontrados nos pacientes classificados nas categorias branca (2,57) e vermelha (2,06). Pacientes com queixas vasculares, endócrinas, neurológicas, cardiológicas ou com problemas em dispositivos, e os que realizaram tomografia de crânio apresentaram alto índice de comorbidade. Ademais, os internados, transferidos ou que morreram na emergência apresentaram significativamente maiores escores do índice em comparação com os que tiveram alta hospitalar.

**Conclusão:**

O alto índice de comorbidade teve associação com as categorias da classificação de risco, queixas principais e inespecíficas, realização de tomografia de crânio e desfechos dos pacientes na emergência.

## INTRODUCTION

The higher prevalence of Chronic Non-Communicable Diseases (CNCDs) can be explained by the increase in the elderly population or by improvements taking place in health care and in the development of society^(1)^. The increase in CNCD^(2)^ and urban violence leads to greater demand for health services, which have become increasingly saturated and insufficient to meet the needs of the population^(3)^. Inserted in this context and due to the low resolution of the Health Care Network, the emergency department (ED) has been used as one of the main gateways to the health system, both for urgent and emergency care, and for diseases with less clinical severity, including some CNCD^(3–4)^.

With the increase in demand and considering the complexity of the morbidities presented by the patients, the EDs have been looking for strategies for the early identification of clinical deterioration, avoiding the occurrence of adverse events^(5)^. One of the main instruments used for this purpose is the risk classification system (*CR*), internationally known as screening^(3)^. In this regard, the priority assessment tool most used worldwide, which demonstrated the possibility of predicting the patient’s risk level and the mortality in the short term, is the Manchester Triage System (MTS)^(6–7)^.

Although MTS is a strategy for critically ill patients to have priority in care in the ED^(3)^, other methods have been used to predict mortality in the short and long term, such as the measurement of comorbidities. Thus, both disease severity and underlying comorbidities are important predictors of mortality after emergency medical admission^(8)^. In this respect, risk-adjusted hospital mortality data have been an essential parameter for monitoring the quality of hospital care, being considered a traditional indicator of clinical performance^(9)^.

Among the comorbidity measures, the Charlson Comorbidity Index (CCI)^(10)^ is highlighted for its ability to assess the impact of the comorbid burden, to estimate hospital mortality in patients with multiple comorbidities^(11–12)^, and for being considered a prognostic indicator for length of stay and survival factors^(13–14)^.

The CCI is a simple method that can be calculated from data obtained from both medical records and administrative data reviews^(14)^, but studies examining the usefulness of CCI as a predictor of mortality in patients treated in the ED are still scarce. The validity of the CCI and its adaptations have been investigated in many international studies and with several subgroups of diseases, including age-adjusted CCI (ACCI)^(15)^, cancer^(10,15)^, stroke^(16)^, acute coronary artery syndrome^(12)^, chronic heart failure^(11)^, and in patients admitted to intensive care^(14)^. In Brazil, the use of the CCI to adjust health care outcome indicators has been infrequent^(17)^.

The importance of this study is highlighted, as risk adjustment for comorbid diseases can help in the assessment of the performance of a hospital facility^(17)^, to improve patient safety and the prognostic predictions of critically ill individuals^(14)^, as well as to help estimate the clinical outcome^(11)^. However, despite its relevance, it is still an underexplored theme in this country. Thus, the question is: What is the relationship between the ACCI and the MTS risk classification categories, the clinical variables, and the outcomes of patients seen in an ED?

The aim of this study was to exam the association between the ACCI and the categories of *CR*, the clinical aspects, and the outcomes of patients in the ED.

## METHOD

### Design of Study

This is a cross-sectional and analytical study.

### Population

The study population consisted of medical records of patients treated at the adult ED of the *Hospital Geral Roberto Santos* (*HGRS*), located in the city of Salvador (BA), Brazil. In 2012, the hospital implemented MTS in the ED, aiming to organize the flow of patients seeking care based on unscheduled and scheduled care.

To include possible seasonality of diseases occurring throughout the year, data were researched for 12 months. The medical records of patients aged 18 years or more, attended at the *CR* sector from January 1 to December 31, 2015, were included in the study, and incomplete or illegible medical records were excluded.

### Sample Definition

The probabilistic sample was representative, and was determined based on a pilot study, which used records of patients seen at the ED of the *HGRS* in December 2014 to collect the proportions of the classification categories of interest, using the simple random sampling without replacement technique, with 95% confidence and maximum allowable error of 2%, and prevalence of 62% for the green color, resulting in a minimum sample size of 2,160 patients. To select the participants, considering that the filed medical records were organized by day, month, and year, systematic sampling was chosen, in which the first medical record in the box was defined as a random starting point and then, in sequence, one in each six individuals was selected to compose the sample. The sample was expanded to 4,157 medical records, with 533 being excluded for not having a record of the *CR* category, reaching the final sample of 3,624 participants.

### Data Collection

Data were accessed at the Medical and Statistical Archiving Service of the institution itself, through manual consultation of patients’ records, from September 2015 to February 2016, using an instrument prepared by the researchers.

For this study, the following variables were analyzed: age, sex, race/color (used to characterize the sample), *CR* category established in the MTS (red for immediate care; orange for very urgent; yellow for urgent; green for slightly urgent; blue for non-urgent; and white used to classify patients coming from other services to undergo evaluation with specialists or complementary tests due to institutional agreements, and those who were referred by a physician, but with no acute or urgent condition)^(3)^, main complaint, diagnostic tests performed in the ED, CCI, and patient outcome after care provision in the ED (discharge, hospital admission, transfer to another health service, and death).

The main complaint was classified according to the organic systems, being related as neurological, respiratory, digestive, cardiac, genitourinary, vascular, endocrine, skin and appendages, mental, ophthalmological, otolaryngological, dental, immunological, and non-specific (those that could not be associated with a specific organ system, such as pain, external causes, intoxication, general malaise, device problems, and others).

**Chart 1:**
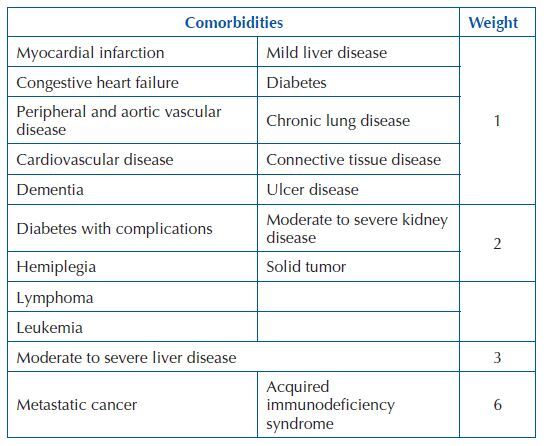
Weight of comorbidities, according to Charlson’s Comorbidity Index.

Comorbidities were defined as health conditions coexisting with the main complaint under investigation. The comorbid burden was calculated using the CCI, which assigns weights of 1, 2, 3 and 6 to each of the existing comorbidities and whose final score is obtained by the sum of these weights^(10,15)^ (Chart 1).

We chose to use the ACCI in the association between the comorbid burden and the MTS *CR* categories, complaints, tests, and outcome, as this index incorporates age into the CCI to predict mortality and survival. The result is established by the sum of the weights of the comorbidities plus a score for each 10-year period, from 50 years onwards: 50 to 59 years old (1 point), 60 to 69 years old (2 points), 70 to 79 years old (3 points) and 80 or more (4 points)^(15)^.

For ACCI interpretation, the following scores were considered: zero (no comorbid burden), ≥ 1 to ≤ 2 (low comorbidity rate/low mortality risk) and > 2 (high comorbidity rate/high mortality risk)^(10)^. The higher the ACCI score, the lower the estimated 10-year survival of patients, that is, scores 1, 2, 3, 4, 5, 6 and ≥ 7 corresponding to survival of 96%, 90%, 77%, 53%, 21%, 2% and 0%, respectively^(10,18)^.

### Data Analysis and Treatment

Data were stored in the software Windows Excel and, for processing and statistical analysis, the software Statistical Package for Social Science (SPSS), version 23, was used. Descriptive analysis was performed by calculating mean, standard deviation, median, minimum, and maximum. The frequency and percentage of categorical variables were calculated. For the association of ACCI with the *CR* categories, main and nonspecific complaint, as well as clinical outcome, analysis of variance (ANOVA) was used. To compare the ACCI score with diagnostic tests, Student’s t-test was used. The significance level considered was 5% (p value < 0.05).

### Ethical Aspects

The study was approved by the Research Ethics Committee (*CEP*) of the Universidade Federal de São Paulo, with the consent of the *CEP* of the Universidade Federal do Recôncavo da Bahia, under opinion no. 773.010/2014, and followed the recommendations of resolution 466/12, of the National Health Council. Considering that patients’ data collection was carried out through medical records, not causing any kind of interference in the ED and harm to the patient, CEP exempted them from signing the Free Informed Consent Term.

## RESULTS

Data from 3,624 patients seen at the *CR* were included. The age ranged from 18 to 114 years, with a mean of 48.4 ± 18.7 years, and there was a predominance of females (51.8%), brown skin (94.4%; n = 2,751), classified in the yellow category risk (31.5%), and with discharge as outcome (87.5%).

Among the patients studied, 1,227 (33.9%) had comorbidities according to the Charlson classification, 919 (25.4%) had clinical condition weight 1, with 414 (11.4%) having uncomplicated diabetes, 249 (6.9%) cerebrovascular disease, 90 (2.5%) peripheral vascular and aortic disease, 50 (1.4%) ulcer disease, 30 (0.8%) dementia/Alzheimer, 25 (0.7%) acute myocardial infarction, 24 (0.7%) congestive heart failure, 20 (0.5%) chronic lung disease, 15 (0.4%) mild liver disease, and two (0.1%) rheumatologic disease.

Among the comorbid diseases weight 2, 85 (2.3%) had moderate to severe kidney disease, 76 (2.1%) diabetes with complications, 51 (1.4%) tumor, 26 (0.7%) hemiplegia/paraplegia, and two (0.1%) patients had leukemia/lymphoma. Moderate to severe liver disease, classified as comorbid disease weight 3, was identified in 42 (1.2%) patients. The comorbidities weight 6 were acquired immunodeficiency syndrome/human immunodeficiency virus and metastatic solid tumor, with 18 (0.5%) and 8 (0.2%) patients, respectively.

In this study, associations were performed using ACCI. Most patients (1,946; 53.7%) had a mean ACCI score of ≥1, with 14.8% showing ACCI of 1, and a chance of survival of 96%; 12.8% had a score of 2, with a survival rate of 90%; 10.4% had a score of 3 and a survival rate of 77%; 8.6% reached score 4 and 53% survival; 3.6% had a score of 5 and 21% survival; for 2.2%, the score was 6, and survival 2%; and only 1.3% had a score ≥7, with a survival rate of 0%. A high comorbidity index (ACCI > 2) was identified in 26.1% of the sample.

The results showed statistical significance in the association of ACCI with the categories of *CR* established by the MTS. Patients with red risk and those in the white category had higher mean scores when compared to the orange, yellow, green, and blue categories (p < 0.0001) (Table 1).

**Table 1.** Age-adjusted Charlson Comorbidity Index by Manchester Triage System risk classification categories – Salvador, BA, Brazil, 2015.

	ACCI				p value*
	N	Mean (SD)	Median	Minimum–Maximum	
**Risk classification**					<0.0001
Red	143	2.06 (2.19)	2	0–10	
Orange	769	1.59 (1.89)	1	0–10	
Yellow	1.142	1.48 (1.82)	1	0–11	
Green	1.004	1.07 (1.46)	0	0–8	
Blue	298	1.14 (1.44)	0	0–6	
White	268	2.57 (2.09)	2	0–9	
Total	3.624	1.47 (1.8)	1	0–11	

* Analysis of variance. ACCI: Age-adjusted Charlson Comorbidity Index; SD: standard deviation.

Patients with vascular, endocrine, neurological, and cardiologic complaints had, on average, higher ACCI scores when compared to the other main complaints (p < 0.0001). There was a significant difference between ACCI and nonspecific complaints. Patients with device problems had higher mean ACCI scores than those who had other nonspecific complaints (Table 2).

**Table 2.** Age-adjusted Charlson Comorbidity Index due to complaints of patients seen in the emergency department – Salvador, BA, Brazil, 2015.

	ACCI				p value^‡^
	N	Mean (SD)*	Median	Minimum–Maximum	
**Main complaint**					<0.0001
Nonspecific	1,904	1 (1.48)	0	0–11	
Neurological	487	2.41 (2.05)	2	0–9	
Skin and appendages	331	1.74 (1.69)	2	0–7	
Digestive	307	1.75 (1.96)	1	0–10	
Respiratory	128	1.99 (2)	1	0–8	
Genitourinary	127	1.71 (1.79)	2	0–9	
Cardiologic	77	2.09 (1.94)	2	0–9	
Vascular	76	3.22 (2.04)	3	0–9	
Otolaryngological	45	1 (1.65)	0	0–7	
Immunological	41	1.07 (1.79)	0	0–6	
Endocrine	39	3.05 (1.86)	3	0–7	
Dental	32	0.69 (1.51)	0	0–6	
Mental	24	1.13 (1.78)	0	0–6	
Ophthalmologic	6	0.17 (0.41)	0	0–1	
**Non-specific complaint**					<0.0001
Pain	1,236	1.01 (1.45)	0	0–11	
External causes	354	0.71 (1.23)	0	0–6	
General malaise	163	1.53 (1.79)	1	0–6	
Exogenous intoxication	110	0.38 (0.94)	0	0–7	
Device problems^†^	21	3.38 (1.99)	4	0–8	
Others	20	1.4 (2.01)	0.5	0–6	

* SD: standard deviation; ^†^ Enteral tube, urinary catheter, hemodialysis catheter; ^‡^ Analysis of variance. ACCI: Age-adjusted Charlson Comorbidity Index; SD: standard deviation.

There was a difference between patients who underwent diagnostic tests and those who did not use the ACCI (p < 0.0001). Patients who underwent electrocardiogram (ECG), laboratory tests, digestive endoscopy, and head tomography had a significantly higher mean ACCI score when compared to those who did not undergo the test. Patients who underwent ultrasonography had a significantly lower mean ACCI score when compared to those who did not undergo the test. Those who underwent head tomography had a mean ACCI score > 2, indicating a high risk of mortality (Table 3).

**Table 3.** Age-adjusted Charlson Comorbidity Index according to diagnostic tests performed by the patients seen in the emergency department – Salvador, BA, Brazil, 2015.

	ACCI				p value*
	N	Mean (SD)	Median	Minimum–Maximum	
**Diagnostic tests**					
Yes	1,335	1.73 (2)	1	0–11	<0.0001
No	2,289	1.31 (1.65)	1	0–9	
**Type of test**					
**Electrocardiogram**					
Yes	174	1.91 (1.95)	1	0–7	0.0024
No	3,450	1.44 (1.79)	1	0–11	
**Radiography**					
Yes	231	1.68 (2.01)	1	0–10	0.0948
No	3,393	1.45 (1.78)	1	0–11	
**Laboratory**					
Yes	547	1.7 (2.1)	1	0–11	0.0037
No	3,077	1.43 (1.73)	1	0–9	
**Head CT**					
Yes	535	2.16 (2.06)	2	0–9	<0.0001
No	3,089	1.35 (1.72)	1	0–11	
**Endoscopy**					
Yes	154	1.77 (1.81)	1	0–6	0.0338
No	3,470	1.45 (1.8)	1	0–11	
**Ultrasound**					
Yes	129	0.84 (1.43)	0	0–7	<0.0001
No	3,495	1.49 (1.81)	1	0–11	

*Student’s t-test. ACCI: Age-adjusted Charlson Comorbidity Index; SD: standard deviation; CT: computed tomography.

Hospital admission, transfer, and death had significantly higher mean ACCI scores (greater than 2) than those who were discharged (Table 4).

**Table 4.** Age-adjusted Charlson Comorbidity Index by outcome of patients seen in the emergency department – Salvador, BA, Brazil, 2015.

	ACCI				p value
	N	Mean (SD)*	Median	Minimum–Maximum	
**Outcome**					
Discharge	3171	1.33 (1.71)	1	0–11	<0.0001*
Admission	83	2.51 (2.22)	2	0–8	
Transfer	275	2.42 (1.98)	2	0–8	
Death	95	2.42 (2.34)	2	0–10	
Total	3,624	1.47 (1.8)	1	0–11	

*Analysis of variance. ACCI: Age-adjusted Charlson Comorbidity Index; SD: standard deviation.

## DISCUSSION

The patients included in this study were mostly women, with a mean age of 48.4 years, classified in the yellow category (urgent) and with discharge as outcome, results that are consistent with those of other international^(19)^ and national^(20–21)^ retrospective studies.

In the ED, *CR* is a formal process for the immediate assessment of all patients seeking emergency care. Such an assessment shall be systematic, and all information shall be gathered to provide a complete picture of the clinical situation. Among the data collected during *CR*, the recording of the patients’ previous morbid past is recommended^(3)^. In this study, the comorbid diseases that make up the most prevalent CCI were diabetes mellitus and cardiovascular disease. These clinical conditions determine mortality risk when compared to other patients without these comorbidities.

Scientific evidence indicates that diabetes, besides being a public health problem, is a globally recognized predictor of mortality, not only for the risk of cardiovascular diseases, but also for several other associated disorders^(12,22)^. These findings draw attention to the constant need to identify and better prevent the multisystem consequences of diabetes^(12)^. In contrast, stroke also continues to be one of the reasons for hospitalization and mortality^(16)^.

The first research to adapt CCI to a large healthcare database in France recommends performing risk adjustment using the ACCI to predict mortality^(23)^. Age equal or above 50 years is considered, together with comorbidity, an aggravating factor in death prediction^(15)^. In Brazil, a study carried out with administrative data from the Brazilian Public Health System (*SUS*) also showed that the effect of the age variable gained greater weight due to the age distribution of the population studied, in which 75.9% of the patients were over 50 years^(17)^. Hence the importance of including age in the assessment of patients upon admission to the ED, as it can influence with survival.

In the individual analysis of the ACCI score, more than half of the sample studied had a score ≥ 1, indicating that most patients had some risk of mortality. However, a high comorbidity/ high mortality index (ACCI > 2) was identified in 26.1% of the sample. Few studies have evaluated ACCI scores in patients with acute illness in the ED. International studies carried out with administrative data from acute admissions identified that less than half (45%) of the patients had an ACCI score of one or more^(24)^ and a less common occurrence for the high rate of comorbidity; only 17.9% of patients had ACCI ≥ 10^(8)^, partially corroborating the findings of this investigation. Another study^(25)^ showed a prevalence of 12.7% for an ACCI score equal to 2, which is similar to what was found in this investigation.

Furthermore, the use of ACCI can also be applied to assess the patients’ estimated 10-year survival^(15,18)^. In this study, there was a less common occurrence of patients with an ACCI score ≥ 4, with an estimated survival of 53% or less.

The results found in this investigation demonstrate that the use of ACCI, despite lacking validation studies for patients classified by the MTS, was able to predict mortality and detect patients with more urgent conditions. Although it was not possible to establish a causal relationship, the highest mean ACCI score was related to the high severity category (red), when compared to the lower severity levels (yellow, green and blue) established in the MTS.

However, it should be noted that patients classified in the white category also presented, on average, high ACCI scores, when compared to the other *CR* categories. This fact can be explained by the characteristic of the hospital studied, which is qualified as a reference center for highly complex care in neurology, vascular surgery, digestive hemorrhage, among other specialties, and receives scheduled patients for diagnostic evaluation and confirmation^(26)^ – especially patients with cerebrovascular diseases who have other associated comorbid diseases, and among the most frequent, diabetes with complications is highlighted^(16)^.

This study showed that patients with vascular and endocrine complaints were significantly associated with higher ACCI scores (p < 0.001), when compared to the other main complaints, with means of 3.22 and 3.05, respectively, which corresponds to a high risk of mortality. This finding can be explained by the high prevalence of diabetes and its complications. A previous study showed that hyperglycemia is considered one of the most common endocrine emergencies in the ED, being associated with mortality and inadequate outpatient treatment for diabetes^(22)^.

The results also demonstrated a high risk of mortality for patients seen with neurological and cardiologic complaints, with a mean ACCI score of 2.41 and 2.09, respectively. Recent studies have investigated the comorbid burden in patients with a range of cardiovascular diseases. The high rate of comorbidity was associated with a significant increase in the risk of mortality in patients with underlying coronary heart disease, heart failure, and stroke^(27)^. A cohort of patients with heart failure found a mean CCI score of 6,^(11)^ and another study showed that the presence of three or more comorbidities was related to a 27% mortality in patients with acute coronary syndrome^(12)^. Thus, scientific evidence indicates that CNCDs, such as cardiovascular diseases, cancer, diabetes and chronic respiratory diseases, lead to repeated hospitalizations in the ED^(25)^ and account for 70% of deaths worldwide^(2)^.

The highest ACCI score was associated with patients with device problems, with a mean of 3.38. This is an unspecific complaint as it was not related to a specific organ system. However, most patients were elderly and all had comorbid diseases, which may explain the high rate of comorbidity. In this group, the reason for the care in the ED was mainly related to obstruction of the enteral tube, urinary catheter or hemodialysis, without acute decompensation of the chronic disease, and this fact may be associated with low resolution in Primary Care focused on this type of care, causing patients to seek care in hospitals. In this study, the low rate of comorbidity was identified in the other nonspecific complaints. The high prevalence of pain is highlighted, corroborating the findings of another national study^(20)^.

Although the CCI measure was developed to predict long-term hospital mortality, it has been increasingly used by hospital systems in real time to identify high-risk patients and guide resources allocation^(28)^. In this study, it was possible to associate ACCI with the use of diagnostic resources. The highest average ACCI score was related to the type of exam; patients who underwent head CT had a mean ACCI score greater than 2, showing a high rate of comorbidity. This finding can be partially explained by the typical characteristics of individuals who habitually used this diagnostic resource, who were older patients with neurological problems and with associated comorbid diseases, such as stroke and hemiplegia.

The performance of ECG, laboratory tests, and digestive endoscopy were also associated with higher scores when compared to those who did not undergo the tests. The national and international literature did not clearly demonstrate the association between comorbid burden and the use of diagnostic tools, but a French study found that, as the ACCI increases, the total annual costs also increase significantly (p < 0.001)^(29)^.

The number and severity of coexisting diseases are an important predictor of complications and unfavorable results^(17)^. A recent survey found that the high severity score of acute illnesses and comorbidities were predictors of hospital mortality within 30 days and common in emergency medical admissions^(8)^. Another multicenter study, carried out in a Danish ED, used the CCI as a marker of the chronic burden of comorbidity and showed that patients with acute hospitalization have a much higher risk of dying compared to the general population^(25)^. These data corroborate our findings, in which the highest mean ACCI scores were identified in patients who required hospitalization, were transferred to other services, and died. In contrast, an American cohort of hospitalized elderly patients examined the prognostic value of CCI in predicting short-term clinical outcomes and found that CCI is a moderate predictor of hospital mortality and a poor predictor of other outcomes such as length of stay and readmissions in 30 days – relevant indicators for administrative health practices^(28)^.

Understanding that ACCI can be automatically generated by the patient registration system, and its score may be able to predict the progression of diseases in patients treated at the ED, the importance of using this indicator as a necessary tool for managers, formulators of policies, and health researchers evaluate the results of the health care provided^(13)^ is highlighted. In addition, ACCI can support health actions aimed at preventing and controlling chronic diseases. In this regard, patients treated in the ED, who have a high comorbid burden and, consequently, a high risk of mortality, need special attention in the care provided, to reduce premature deaths after hospitalization.

The number of short-term deaths occurring in the ED is associated with the severity category assessed through the application of the MTS^(7)^ and with higher mean ACCI scores found in this investigation. However, further research is needed to determine whether the use of a comorbidity index associated with MTS can increase the sensitivity and specificity of the protocol in predicting death.

This study had as limitation being carried out in a single center and having characteristics that are inherent to studies conducted based on paper records, such as illegibility and incompleteness of the information recorded by health professionals. It should be noted that, in this study, 533 medical records that did not have information on the color of the *CR* were lost, which certainly did not affect the results of this investigation, due to the large sample size. The losses reveal that the non- computerized system allows for greater chances of omissions in the data records, and an analysis on the subject is pertinent to establish improvements.

## CONCLUSION

ACCI showed a significant relationship with the *CR* categories established by the MTS, main or nonspecific complaint, diagnostic tests performed, and emergency outcomes. High risk of mortality (ACCI > 2) was identified in patients classified in the red and white MTS categories, with vascular, endocrine, neurological, cardiologic complaints, or who had device problems, who underwent head tomography, and in those who were hospitalized, transferred, or died in the emergency department.

## Financial support

This work was carried out with the support of the Coordenação de Aperfeiçoamento de Pessoal de Nível Superior – Brazil (CAPES) – Financing Code – 001.

## References

[1] Van Oostrom SH, Gijsen R, Stirbu I, Korevaar JC, Schellevis FG, Picavet HC (2016). Time trends in prevalence of chronic diseases and multimorbidity
not only due to aging: Data from general practices and health
surveys. PLoS One..

[2] World Health Organization (2017). Noncommunicable Diseases Progress Monitor 2017.

[3] Mackway-Jones K, Marsden J, Windle J (2014). Emergency Triage: Manchester Triage Group [Internet].

[4] Siochetta TM, Silva A, Beuren AC (2019). Baixa resolutividade na rede de atenção à saúde: Um problema
vigente. Revista Saúde Integrada [Internet].

[5] Schipper EM (2017). Acute medical units, more capacity without increasing
resources. Eur J Intern Med..

[6] Zachariasse JM, Seiger N, Rood PPM, Alves CF, Freitas P, Smit FJ (2017). Validity of the Manchester Triage System in emergency care: A
prospective observational stydy. PLoS One..

[7] Martins HM, Cuña LM, Freitas P (2009). Is Manchester (MTS) more than a triage system? A study of its
association with mortality and admission to a large Portuguese
hospital. Emergency Medicine Journal..

[8] Conway R, Byrne D, O’Riordan D, Silke B (2020). Comparative influence of acute illness severity and comorbidity
on mortality. Eur J Intern Med..

[9] Cordeiro P, Martins M (2018). Hospital mortality in older patients in the Brazilian Unified
Health System, southeast region. Rev Saude Publica..

[10] Charlson ME, Pompei P, Ales KL, MacKenzie CR (1987). A new method of classifying prognostic comorbidity in
longitudinal studies: Development and validation. J Chronic Dis..

[11] Shuvy M, Zwas DR, Keren A, Gotsman I (2020). The age-adjusted Charlson comorbidity index: A significant
predictor of clinical outcome in patients with heart failure. Eur J Intern Med..

[12] Sanchis J, Soler M, Núñez J, Ruiz V, Bonanad C, Formiga F (2019). Comorbidity assessment for mortality risk stratification in
elderly patients with acute coronary syndrome. Eur J Intern Med..

[13] Austin SR, Wong YN, Uzzo RG, Beck JR, Egleston BL (2015). Why summary comorbidity measures such as the Charlson Comorbidity
Index and Elixhauser score work. Med Care..

[14] Stavem K, Hoel H, Skjaker SA, Haagensen R (2017). Charlson comorbidity index derived from chart review or
administrative data: agreement and prediction of mortality in intensive care
patients. Clin Epidemiol..

[15] Charlson M, Szatrowski TP, Peterson J, Gold J (1994). Validation of a combined comorbidity index. J Clin Epidemiol..

[16] Hall RE, Porter J, Quan H, Reeves MJ (2019). Developing na adapted Charlson comorbidity index for ischemic
stroke outcome studies. BMC Health Serv Res..

[17] Martins M, Travassos C, Noronha JC (2001). Brazilian Hospital Database System as risk adjustment in
performance indicators. Rev Saude Publica..

[18] Charlson M Charlson Comorbidity Index – MDCalc.

[19] Martins JCA, Guedes HM, Souza CC, Chianca TCM (2018). Association between vital signs and Manchester Triage System: A
retrospective observational study. Online Brazilian Journal of Nursing..

[20] Oliveira GN, Vancini-Campanharo CR, Lopes MCBT, Barbosa DA, Okuno MFP, Batista REA (2016). Correlation between classification in risk categories and
clinical aspects and outcomes. Rev Lat Am Enfermagem..

[21] Mendes TJM, Silveira LM, Silva LP, Stabile AM (2018). Association between reception with risk classification, clinical
outcome and the Mews Score. REME.

[22] Echouffo-Tcheugui JB, Garg R (2017). Management of hyperglycemia and diabetes in the emergency
department. Curr Diab Rep..

[23] Bannay A, Chaignot C, Blotière PO, Basson M, Weill A, Ricordeau P (2016). The best use of the charlson comorbidity index with electronic
health care database to predict mortality. Med Care..

[24] Vest-Hansen B, Riis AH, Sorensen HT, Christiansen CF (2014). Acute admissions to medical departments in Denmark: Diagnoses and
patient characteristics. Eur J Intern Med..

[25] Flojstrup M, Henriksen DP, Brabrand M (2017). An acute hospital admission greatly increases one year mortality
– Getting sick and ending up in hospital is bad for you: A multicentre
retrospective cohort study. Eur J Intern Med..

[26] Secretaria de Saúde do Estado da Bahia Hospital Geral Roberto Santos.

[27] Rashid M, Kwok CS, Gale CP, Doherty P, Olier I, Sperrin M (2017). Impact of comorbid burden on mortality in patients with coronary
heart disease, heart failure, and cerebrovascular accident: a systematic
review and meta-analysis. Eur Heart J Qual Care Clin Outcomes..

[28] Sinvani L, Kuriakose R, Tariq S, Kozikowski A, Patel V, Smilios C (2019). Using Charlson comorbidity index to predict short-term clinical
outcomes in hospitalized older adults. J Healthc Qual..

[29] Charlson M, Wells MT, Ullman R, King F, Shmukler C (2014). The Charlson comorbidity index can be used prospectively to
identify patients who will incur high future costs. PLoS One..

